# Application of the ex-Gaussian function to the effect of the word blindness suggestion on Stroop task performance suggests no word blindness

**DOI:** 10.3389/fpsyg.2013.00647

**Published:** 2013-09-20

**Authors:** Benjamin A. Parris, Zoltan Dienes, Timothy L. Hodgson

**Affiliations:** ^1^Psychology Research Centre, School of Design, Engineering and Computing, University of BournemouthPoole, UK; ^2^School of Psychology and Sackler Centre for Consciousness Science, University of SussexBrighton, UK; ^3^School of Psychology, University of LincolnLincoln, UK

**Keywords:** suggestion, Stroop, ex-Gaussian, post-hypnotic, response competition, hypnosis

## Abstract

The aim of the present paper was to apply the ex-Gaussian function to data reported by Parris et al. (2012) given its utility in studies involving the Stroop task. Parris et al. showed an effect of the word blindness suggestion when Response-Stimulus Interval (RSI) was 500 ms but not when it was 3500 ms. Analysis revealed that: (1) The effect of the suggestion on interference is observed in μ, supporting converging evidence indicating the suggestion operates over response competition mechanisms; and, (2) Contrary to Parris et al. an effect of the suggestion was observed in μ when RSI was 3500 ms. The reanalysis of the data from Parris et al. (2012) supports the utility of ex-Gaussian analysis in revealing effects that might otherwise be thought of as absent. We suggest that word reading itself is not suppressed by the suggestion but instead that response conflict is dealt with more effectively.

## Introduction

The Stroop task is a selective attention task that requires participants to identify the color of the font in which a word is presented, whilst ignoring the word itself (Stroop, 1935; see MacLeod, 1991, for a review). Performance in the task is indexed by measures of interference and facilitation which are thought to result from the automatic processing of the irrelevant dimension of the Stroop stimulus. When the written word is incongruent with the font color (e.g., *green* written in red), the time it takes to identify the color is increased relative to a baseline control condition (e.g., *flower* written in red), a difference known as Stroop interference. When the color and word are congruent (e.g., *red* written in red) the time it takes to identify the color is decreased relative to the baseline control condition, a difference known as Stroop facilitation. The Stroop effect is one of the most robust in cognitive psychological research and has been referred to as the “gold standard” of measures of attention (MacLeod, 1992). Most extant theories argue that Stroop interference is the result of response competition, whilst Stroop facilitation is the result of response convergence (Cohen et al., 1990; Melara and Algom, 2003; Roelofs, 2003; but see MacLeod and MacDonald, 2000; Kane and Engle, 2003 for contrasting views).

A recent, remarkable finding showed that the Stroop effect can be effectively eliminated (Raz et al., 2002). Raz et al. hypnotized their participants and whilst under hypnosis delivered a suggestion indicating that once they were no longer hypnotized they would play a computer game (the Stroop task) and that any stimulus they saw would be made up of “meaningless symbols” and “characters of a foreign language” (to be referred to as the *word blindness* suggestion; Raz et al., 2002). Once they were counted out of hypnosis the experimenter clapped to activate the word blindness post-hypnotic suggestion, which was also the cue for participants to being the Stroop task. The effect was remarkable, resulting in an all-encompassing effect on indices of Stroop task performance, eliminating both interference and facilitation effects. The authors argued that their results were inconsistent with the notion that processes of visual word recognition are automatic and that the post-hypnotic suggestion works via a top-down mechanism that modifies the processing of input words through a means not voluntarily available, rendering the words meaningless.

Recent work indicates that, contrary to Raz et al.'s interpretation, the suggestion does not result in rendering the words meaningless but instead reduces response competition (Augustinova and Ferrand, 2012). Augustinova and Ferrand showed that the word blindness suggestion does not affect semantic-associative interference (e.g., “sky,” associated with the color blue, in yellow); interference that does not involve response competition. The present work applied the ex-Gaussian function to data originally presented by Parris et al. (2012). It has been argued that the μ component of the ex-Gaussian distribution mainly indexes response conflict (Kane and Engle, 2003; Steinhauser and Hübner, 2009) and thus according to the response conflict account of the word blindness suggestion effect, should be uniquely affected by the suggestion.

Numerous subsequent studies have shown that observing a reduction in Stroop interference following the suggestion is replicable (Raz et al., 2003, 2005, 2006, 2007; Raz and Campbell, 2009; Augustinova and Ferrand, 2012; Parris et al., 2012, 2013) and others have shown that similar effects can be observed in other selective attention paradigms such as the Erikson Flanker task (Iani et al., 2006) and the Simon task (Iani et al., 2009). In a fully within-subjects and counterbalanced design Parris et al. (2012) showed that the effect of the suggestion on Stroop task performance is more likely when Response-Stimulus Interval (RSI) is 500 ms compared to when it is 3500 ms (equivalent to that used by Raz and colleagues in their studies). They showed that the suggestion reduced Stroop interference in the short RSI condition (from 54 to 56 ms) but not in the long RSI condition (from 52 to 56 ms), and did not affect Stroop facilitation (see Table 2). Thus, the suggestion effect was shown to be subject to similar temporal constraints as those recently observed on conflict adaptation effects (Egner et al., 2010) and evinced that response to the suggestion involves reactive top-down control processes that persist only if levels of activation can be maintained between trials (as they evidently were in Raz et al.'s original 2002 study). Importantly, aside from the RSI manipulation, the methods employed by Parris et al. were identical to those employed by Raz and colleagues. Parris et al. showed that the RSI effect on the suggestion was not due to time-on-task effects; despite taking longer to complete and therefore requiring sustaining of the suggestion over a long time period, Parris et al. showed that the effect in the short RSI condition was stronger in those participants that completed the long RSI condition first, even though no effect was observed for the same participants when the RSI was long. Their results implied that: (1) the cue given to activate the suggestion (a clap) was not enough to fully activate the suggestion; (2) the suggestion is reactivated on every trial by the presence of the Stroop stimulus; and, (3) maintaining activation of the suggestion is an effortful process.

### The utility of the ex-Gaussian function

As with almost all standard cognitive experimental paradigms that make use of reaction time (RT) as a dependent measure, interference and facilitation effects in studies using the word blindness suggestion were computed from algebraic mean reaction times. However, this often results in information loss since RT distributions typically have a positively skewed unimodal shape. Recently, Balota and Yap (2011) highlighted how distributional analysis procedures, particularly the ex-Gaussian approach, can be used to better understand effects observed in standard cognitive experimental paradigms, since it takes into account the typical shape of RT distributions. The ex-Gaussian distribution is a mathematical convolution of the normal (Gaussian) and exponential distributions and has three parameters which are μ (mu) and σ (sigma), reflecting the mean and standard deviation of the Gaussian distribution, and τ (tau), reflecting the mean and standard deviation of the exponential distribution. Intuitively, distribution shifting is reflected in μ, and distribution skewing is reflected in τ (Heathcote et al., 1991).

One of the first applications of the ex-Gaussian function in experimental psychology was to the Stroop paradigm (Heathcote et al., 1991). Heathcote et al. (1991) had participants perform a vocal Stroop task consisting of incongruent, neutral (a series of x's), and congruent trials with the aim of comparing the standard algebraic mean analysis to the ex-Gaussian analysis of the same data set. Interference, but not facilitation, effects were observed in the standard analysis; indeed, facilitation effects are not always observed, and when they are observed are about half the size of interference effects (MacLeod, 1991). Heathcote et al. also analyzed the standard deviations (SD), revealing an interference effect in both the incongruent and congruent conditions (that is, the SDs for the incongruent and congruent conditions were larger than those for the baseline condition, although the incongruent SD was larger than that for congruent trials indicating a Stroop congruency effect).

Ex-Gaussian analysis of the same data served as a counterpoint to the algebraic mean analysis. Analysis of μ revealed both interference and facilitation effects, contrasting with effects observed in the standard analysis that showed no facilitation effect. σ showed interference, but no facilitation. Revealingly, τ showed interference in both the incongruent and congruent conditions but did not differ for incongruent and congruent trials. Since the algebraic mean is equal to μ + τ, these results show that the lack of facilitation in the algebraic mean data was the result of the increase in skew in the congruent trial RT distribution, which effectively hid the facilitation observed in μ. Thus, congruent words did influence performance, but its effects were masked by a change in shape of the RT distribution. Importantly, these results, replicated by Mewhort et al. (1992), provide an account for the inconsistent or small facilitation effects commonly observed in the Stroop literature.

Heathcote et al.'s results highlight the utility of the ex-Gaussian approach. They noted how the ex-Gaussian distribution should be treated as a descriptive first-order account of response latency since it provides a good description of the data and can show whether skew is systematically affected by a particular condition or is, in contrast, a nuisance variable such as a lapse of attention that is not associated with any particular experimental condition. In Heathcote et al.'s data an interference effect was observed in τ indicating that skew was indeed systematically affected by an experimental manipulation. In contrast, in the individual differences literature τ has been interpreted as a measure of lapses of attention. For example, Spieler et al. (1996) showed that compared to young adults, older adults showed an increase in the tail of the distribution in the incongruent condition, reflecting a population of trials in which the older adults experienced increased interference from the word dimension. Since this effect was specific to incongruent trials they interpreted the data as being consistent with the notion that older adults experience difficulty in inhibiting a conflicting word code or sufficiently focussing on the goal of color naming. Importantly, the lack of a shift in the modal portion of the RT distribution suggested that the effect of ageing was not simply generalized slowing. Moreover, in the same paper a comparison between very mild Dementia of the Alzheimer's Type (DAT) and mild DAT patients in the algebraic mean data revealed a non-significant difference, but analysis of the ex-Gaussian parameters revealed a very large increase in interference in μ in the mild DAT patients accompanied by a very large decrease in interference in τ, thereby offsetting effects in the algebraic mean data.

### Interpretation of ex-Gaussian parameters

Although attempts have been made to attribute cognitive processes to ex-Gaussian parameters (Hohle, 1965; Steinhauser and Hübner, 2009; Roelofs, 2012) some researchers have made repeated exhortations about the perils of such an exercise (Heathcote et al., 1991; Matzke and Wagenmakers, 2009). For example, τ appears to increase with increased conflict (Heathcote et al., 1991), impaired goal maintenance (Spieler et al., 1996; Roelofs, 2010), reduced inhibitory control (Spieler et al., 1996), increased task conflict (Steinhauser and Hübner, 2009; Roelofs, 2012), or increased spatial integration (Spieler et al., 2000; but see Roelofs, 2012) suggesting that it indexes more than one cognitive process. In contrast it has recently been argued that μ mainly indexes response conflict (Kane and Engle, 2003; Steinhauser and Hübner, 2009). However, following Heathcote et al.'s suggestion, the aim of the present experiment was to initially treat the ex-Gaussian distribution as a descriptive first-order account of response latency to investigate whether the word blindness suggestion influences mainly distributional skew, distributional shifting or both.

Application of the ex-Gaussian function to the effect of the word blindness suggestion on Stroop task performance permits a deeper exploration of the effect than algebraic mean analysis. For example, the suggestion effect could result from a shift in the RT distribution, a decrease in skew in the distribution, or both. Few studies have observed a complete elimination of Stroop interference (see Table 1, for a summary of the proportional effects of the suggestion on interference and facilitation across studies reporting replications of the effect) suggesting that interference in μ or τ might be unaffected. The suggestion might also have opposing effects on distributional shifting and skewing. For example, Table 1 reveals a proportionally smaller effect of the suggestion on facilitation than on interference across studies, a finding that is difficult to explain under models of Stroop task performance that assume that interference and facilitation are the result of the same single mechanism (Cohen et al., 1990; Melara and Algom, 2003; Roelofs, 2003); if word reading is disabled, interference and facilitation effects should be affected in tandem (Brown, 2011). Given that facilitation in the algebraic mean data has been shown to be the sum of facilitation in μ and interference in τ (congruent longer than neutral RTs; Heathcote et al., 1991; Mewhort et al., 1992; Spieler et al., 1996), the smaller effect on facilitation could be the result of the suggestion reducing facilitation in μ, but greatly increasing skew in the congruent condition relative to the neutral condition. Such opposing effects would limit the apparent effect of the suggestion on facilitation in the algebraic mean data. Finally, since all studies observing the effect of the suggestion on performance have trimmed the RT data, removing any RTs above or below 3SDs from the mean, it is possible that the suggestion results in increased skew in the incongruent condition (an increase in the number of trials on which there is a slow response), which would be trimmed away and consequently reduce apparent levels of interference. Thus, the application of the ex-Gaussian function to the word blindness suggestion allows a finer-grained analysis of the effect.

**Table 1:** **Published studies showing significant effects of the suggestion on the classic Stroop interference effect using highly hypnotizable individuals (RTs in milliseconds, Interference = Incongruent − Neutral, Congruent = Neutral − Congruent)**.

**Study**	**Type of suggestion**	***N***	**Interference without suggestion**	**Interference with suggestion**	**Reduction as a proportion of interference without suggestion (%)**	**Facilitation without suggestion**	**Facilitation with Suggestion**	**Reduction as a proportion of facilitation without suggestion (%)**
Raz et al., 2002	Post-hypnotic	16	112	−2	100	45	7	84.5
Raz et al., 2003	Post-hypnotic	6	102	19	81.4	33	3	90.9
Raz et al., 2005	Post-hypnotic	8	90	3	96.7	30	33	0
Raz et al., 2006	Post-hypnotic	13	94	53	43.6	38	28	26.3
	Non-hypnotic	12	78	43	44.9	38	33	13.2
Raz et al., 2007	Post-hypnotic	49	78	6	92.3	40	10	75
Augustinova and Ferrand, 2012 (Experiment 2)	Non-hypnotic	15	146	114	21.9	38	30	21.1
Parris et al., 2012, Short (500 ms) Response Stimulus Interval condition	Post-hypnotic	19	54	6	88.9	15	19	0
		Average	94.3	30.5	67.7	34.6	20.4	40

The table shows facilitation effects are not as frequently influenced by the suggestion, nor are they influenced to the same extent.

## Materials and methods

For a full reporting of Materials and Methods please refer to Parris et al. (2012).

## Results

### Analysis of the non-trimmed algebraic mean data from Parris et al. (2012)

In Parris et al.'s report, the data were trimmed such that any RTs 3SDs either side of the mean were removed before analysis. However, given the purpose of the present reanalysis, and following the recommendations of Heathcote et al. (1991) and Ulrich and Miller. (1994; see also Roelofs, 2012), the RT data presented here were not trimmed. Using trimming to correct a skewed distribution may discard valuable information, especially if the skew itself is the topic of investigation. Table 2 shows a comparison of the trimmed and non-trimmed mean data. Since Parris et al. presented trimmed data we first analyzed the non-trimmed data, which revealed the same pattern of results as those observed with the trimmed data. The ex-Gaussian function was then applied to the data.

**Table 2:** **A comparison of trimmed and non-trimmed means in milliseconds (with standard deviations in brackets) from Parris et al. (2012) as a function of Stroop stimulus, Response-Stimulus Interval, and presence or absence of suggestion**.

	**Response-stimulus interval**	**Suggestion present**		**Suggestion absent**	
		**500 ms**	**3500 ms**	**500 ms**	**3500 ms**
Trimmed with 3 SDs either side of the mean removed (from Parris et al., 2012)	Incongruent	681 (101)	754 (128)	761 (131)	788 (132)
	Neutral	674 (93)	698 (119)	708 (95)	736 (120)
	Congruent	655 (93)	685 (116)	692 (104)	716 (111)
	Interference	6	56	54	52
	Facilitation	19	12	15	20
Not trimmed (used in the present ex-Gaussian analysis)	Incongruent	686 (113)	762 (144)	774 (163)	801 (142)
	Neutral	677 (103)	701 (127)	712 (104)	745 (123)
	Congruent	657 (94)	688 (122)	697 (113)	719 (117)
	Interference	9	60	62	56
	Facilitation	20	14	15	26

As is evident from the comparison of the pattern of the means in Table 2, the effects reported in Parris et al. remain in the non-trimmed data indicating that, at the level of algebraic mean analysis, there was little or no data loss as a result of the trimming. This was confirmed in a 3 (Word Type: Incongruent/Neutral/Congruent) × 2 (Post-Hypnotic Suggestion: Present/Absent) × 2 (Response Stimulus Interval: 500 ms/3500 ms) repeated measures ANOVA. The main effects of Suggestion and Word type were significant (*p*'s < 0.005), as were the two-way interactions between Suggestion and Word Type, and RSI and Word type (*p*'s < 0.05). The main effect of RSI and the two-way interaction between Suggestion and RSI were not significant (*p*'s > 0.05). Critically, the three-way interaction was significant where *F*_(2, 36)_ = 3.767, *p* < 0.03, η^2^ = 0.173. These results perfectly match the pattern of significant and non-significant effects observed in Parris et al. showing that entering all data points into the analysis had little substantive effect on observed effects.

### Ex-Gaussian analysis

Despite evidence of no loss of effects at the level of the algebraic means reported above, data trimming could result in loss of information at the level of distributional analysis; hence we inputted the non-trimmed data into the ex-Gaussian analysis. Estimated values for μ, σ, and τ were reached after no more than 40 iterations using QMPE (Heathcote et al., 2004) and using N-3 (45) quantiles, where *N* is the number of trials per cell. Whilst the RTs for each individual were assumed to have an ex-Gaussian distribution, the means entered for each of the ex-Gaussian parameters were assumed to be normally distributed and were thus subjected to a 3 (Word Type: Incongruent/Neutral/Congruent) × 2 (Post-Hypnotic Suggestion: Present/Absent) × 2 (Response Stimulus Interval: 500 ms/3500 ms) repeated measures ANOVA. μ, σ, and τ were entered independently. Inspection of Q-Q plots (see Figure 2) revealed an acceptable goodness-of-fit of the predicted to observed quantiles. See Table 3 for values of μ, σ, and τ as a function of condition.

**Table 3:** **Estimates of the ex-Gaussian parameters (in ms; SDs in brackets) μ, σ, and τ as a function of condition**.

	**Response-stimulus interval**	**Suggestion present**		**Suggestion absent**	
		**500 ms**	**3500 ms**	**500 ms**	**3500 ms**
μ	Incongruent	510 (70)	590 (101)	565 (112)	628 (127)
	Neutral	541 (73)	578 (117)	544 (96)	606 (116)
	Congruent	514 (78)	570 (87)	540 (88)	587 (114)
	Interference	−32	12	20	22
	Facilitation	27	9	3	18
σ	Incongruent	78 (44)	94 (58)	106 (73)	113 (85)
	Neutral	82 (32)	110 (76)	72 (30)	99 (44)
	Congruent	72 (46)	93 (60)	74 (32)	88 (33)
	Interference	−3	−16	34	14
	Facilitation	10	17	−1	10
τ	Incongruent	179 (92)	161 (64)	212 (123)	175 (109)
	Neutral	136 (100)	117 (44)	167 (100)	133 (88)
	Congruent	144 (79)	110 (66)	156 (93)	139 (73)
	Interference	42	44	45	42
	Facilitation	−7	7	11	−6

**Figure 1:**
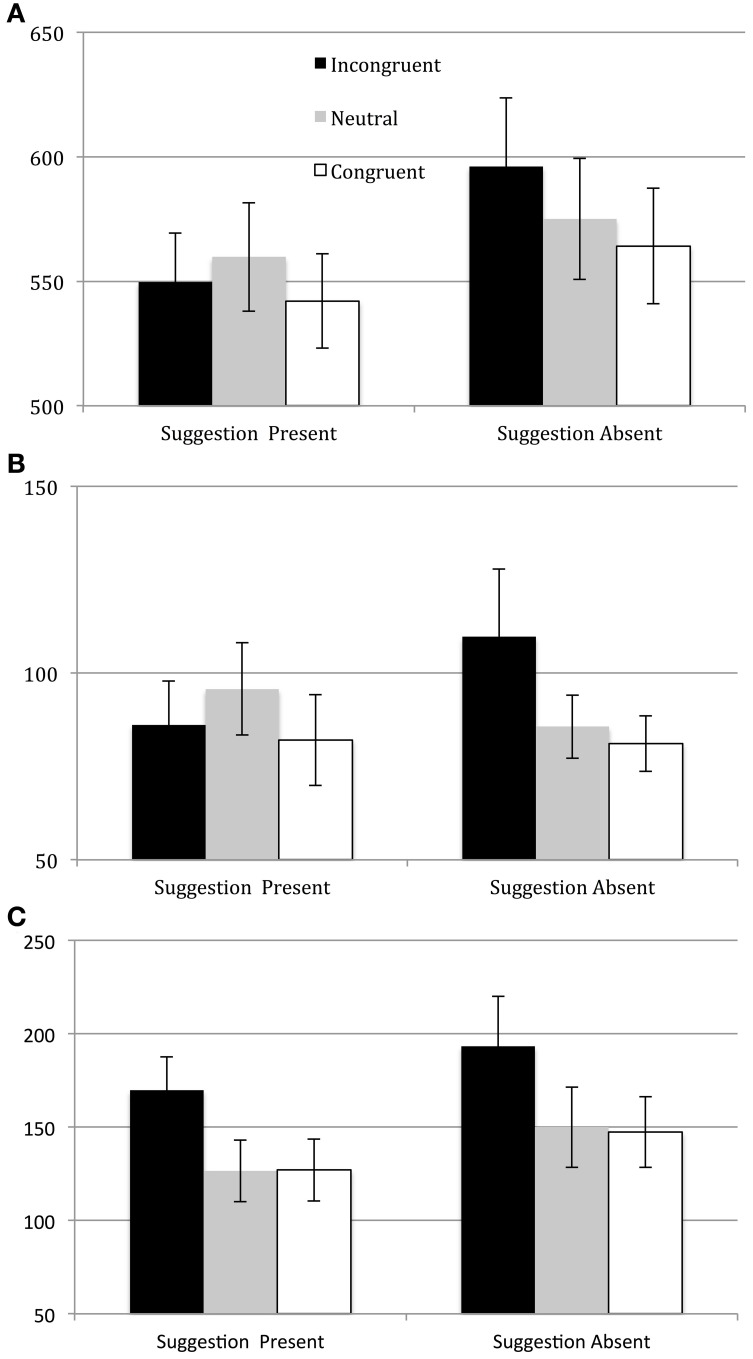
**(A)** Values for μ **(A)**, σ **(B)**, and τ **(C)** in milliseconds as a function of condition (collapsing across the Response-Stimulus Interval manipulation).

Analysis of the μ parameter revealed that the only significant interaction was the two-way interaction between Suggestion and Word Type where *F*_(2, 36)_ = 3.757, *p* < 0.05, partial η^2^ = 0.173 (see Table 4). For all other interactions *p*'s > 0.15. See Figure 1 for values of μ as a function of condition (collapsing across RSI). To explore the two-way interaction we compared the magnitudes of both Stroop interference and Stroop facilitation in the two levels of the post-hypnotic suggestion factor, collapsing across RSI. The results showed that Stroop interference was significantly larger in the Suggestion Absent condition than in the Suggestion Present condition where *F*_(1, 18)_ = 5.062, *p* < 0.05, partial η^2^ = 0.219, but that Stroop facilitation was not significant, *F*_(1, 18)_ = 0.650, *p* > 0.4. These results show that, contrary to the analysis of the algebraic mean data in Parris et al. (2012), the influence of the post-hypnotic suggestion was not strongly modulated by RSI in μ. Indeed, analysis of each RSI condition separately revealed an effect of the suggestion in both the short [*F*_(2, 36)_ = 5.913, *p* < 0.01, partial η^2^ = 0.247] and the long [*F*_(2, 36)_ = 3.377, *p* < 0.05, partial η^2^ = 0.158] RSI conditions.

**Table 4:** **Algebraic means and estimates of μ, σ, and τ for Stroop interference and facilitation (ms) as a function of condition when collapsing across Response-Stimulus Interval**.

	**Post-hypnotic suggestion present**	**Post-hypnotic suggestion absent**	***p*-value from paired-sample *t*-test (and Bayes Factor)**
**INTERFERENCE**			
MRT	34.5	58	*p* < 0.05 (4.92)
μ	−10	21	*p* < 0.05 (4.02)
σ	−14	24	*p* < 0.05 (5.18)
τ	43^**^	43^**^	*p* > 0.9 (0.37)
**FACILITATION**			
MRT	17	20.5	*p* > 0.8 (0.76)
μ	18^*^	11	*p* > 0.4 (1.92)
σ	13.5	4.5	*p* > 0.2 (1.34)
τ	0	3	*p* > 0.7 (1.03)

The final column shows the p-values for Paired-sample t-test comparisons between the Suggestion Present and Suggestion Absent conditions. Bayes Factors are presented within the brackets. A Bayes Factor of 0.33 or lower is strong evidence for the null hypothesis. A Bayes Factor of 3 or above is strong evidence of a difference. Any value in between is inconclusive. Bayes Factors were calculated using a standard error adjusted for small sample size using a uniform with a lower bound of 0 and an upper bound of the size of each coefficient in the Suggestion Absent condition (see Dienes, 2008, 2011).

* p < 0.05;

** p < 0.001.

Analysis of the σ parameter revealed no significant effects, although there was a trend toward a main effect of RSI where *F*_(1, 18)_ = 4.104, *p* = 0.05, η^2^ = 0.186 and a trend toward an interaction between suggestion and word type [*F*_(2, 36)_ = 3.582, *p* = 0.06 (Greenhouse–Geisser), η^2^ = 0.166 (all other *p*'s > 0.115)].

**Figure 2:**
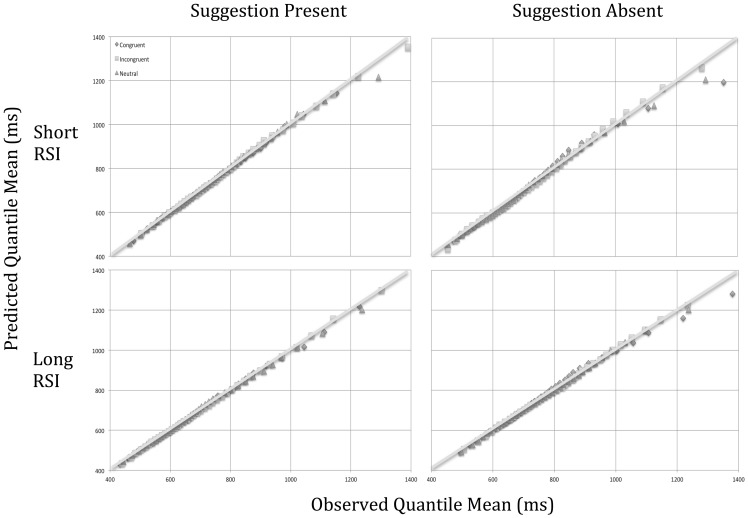
**Q-Q Plots showing goodness-of-fit for the estimated values of the ex-Gaussian parameters**.

Analysis of the τ parameter revealed that the only significant effect was a main effect of Word Type [*F*_(2, 36)_ = 16.503, *p* < 0.001, partial η^2^ = 0.478]. Interference effects were robust in both the suggestion present and suggestion absent conditions (see Table 4). See Figure 2 for values of τ as a function of condition (collapsing across RSI).

### Calculation of bayes factors for key comparisons

Table 4 shows the values of μ, σ, and τ as a function of condition when collapsing across RSI. In the third column of the table, *p*-values for paired-sample *t*-tests are reported along with number in brackets representing Bayes Factors (Dienes, 2008, 2011). For the algebraic mean data and each of the values of μ, σ, and τ we used a Bayes Factor to contrast the theory that the suggestion had some effect with the null hypothesis that the suggestion had no effect. For example, the non-significant two-way interaction between Word Type and Suggestion in τ is consistent with either evidence for no reduction of the interference effect or simply with the absence of evidence for a reduction. We modeled the predictions of the theory of some effect with a uniform between 0 and 43 ms (see Dienes, 2011, Appendix), i.e., any effect was as plausible as any other in the full range (43 ms is the size of the interference effect in the no suggestion condition). The resulting Bayes Factor was 0.37, indicating that the data are inconclusive with regards to whether or not there was an effect of the suggestion on τ (0.33 and below being the cut-off for strong evidence for the null, Jeffreys, 1961; Dienes, 2011). No comparison yielded a Bayes Factor lower than 0.33 indicating that there was no evidence for the null hypothesis in any comparison. Indeed a Bayes Factor between 0.33 and 3 is deemed inconclusive with regards to effects observed meaning that effects of the suggestion on facilitation are also inconclusive in the present data set. In contrast, and complementing the analysis above, comparisons investigating effect of the suggestion on interference in the algebraic mean data, μ and σ revealed Bayes Factors greater than 3 indicating strong evidence for an effect on performance.

### Comparison to previous ex-Gaussian analyses of stroop task performance

We compared our Suggestion Absent, Long RSI condition against the most common finding across all previous studies since the longer RSI is likely to be most similar to these studies given that the start of each trial in their studies was determined by human input and not tightly controlled by computer. Results from studies testing young adults have found the following fairly consistent pattern in the application of the ex-Gaussian function to Stroop performance: μ, σ, and τ increase for incongruent relative to the baseline condition showing a Stroop interference effect in all ex-Gaussian parameters, but both interference and facilitation effects are numerically larger in μ; in contrast, μ decreases and τ increases for congruent trials relative to neutral trials, whilst for σ there is little difference between congruent and neutral trials (Heathcote et al., 1991; Spieler et al., 1996, 2000). In the present data interference and facilitation effects were as follows: Neither interference (22 ms) nor facilitation (18 ms) reached significance in μ (*p*'s > 0.2). Similarly, neither interference (14 ms) nor facilitation (10 ms) were significant in σ (*p*'s > 0.3). In line with previous studies, values for congruent trials were larger than those for neutral trials in τ (by 6 ms). However, this difference did not reach significance (*p* > 0.7). In contrast, interference (42 ms) in τ was significant where *t*_(18)_ = 2.250, *p* < 0.05. Thus, only the finding of interference in τ and a lack of facilitation in σ is consistent with previous studies applying the ex-Gaussian function to Stroop task performance.

Consideration of just one of the methodological differences likely to affect Stroop effects across ex-Gaussian parameters, namely the issue of manual vs. vocal responding, renders differences less important: It is commonly observed that Stroop effects when using vocal responses are double that observed when using manual responses (see MacLeod, 1991, for a review). Indeed there has historically been a debate as to whether or not the same cognitive processes are involved in the Stroop task when responses and manual or vocal (see Glaser and Glaser, 1989; Sugg and McDonald, 1994; Sharma and McKenna, 1998). For example, it has been claimed that there is no semantic processing with a manual response (Sharma and McKenna, 1998; see also Brown and Besner, 2001). To the best of our knowledge there is no study comparing Stroop effects in ex-Gaussian parameters in manual and vocal responses and thus it is unknown what type of effect response mode might have on distributional shifting and skewing. Despite the lack of a study directly comparing the effects of the two response modes on ex-Gaussian parameters, an insight can be gained by considering the results from Steinhauser and Hübner (2009) the only other study of which we are aware that employed a manual response in the Stroop task and subsequently subjected the data to ex-Gaussian analysis. Whilst they observed numerical interference in μ (roughly 50 ms), σ (roughly 20 ms), and τ (roughly 25 ms), they observed no difference between neutral and congruent (their “identical”) trials in μ, and a decrease in σ (roughly 10 ms), and τ (roughly 20 ms) values for congruent trials relative to their xxxx (“univalent”) condition (these comparison are drawn from Steinhauser and Hubner's constant block color naming trials). Analyses of these Stroop effects were not reported and thus it is not known which of these effects reached significance. It is clear from these results that careful experiments are required to pinpoint factors affecting the magnitude of interference and facilitation effects across the ex-Gaussian parameters.

As noted above another key difference between our study and previous studies is that neither Heathcote et al. nor Spieler et al. controlled the length of time between Stroop trials since in both studies the start of each trial was determined by human input from either the participant or experimenter. Moreover, we tested a different, special population of individuals. These differences notwithstanding, the key point to note in the present paper is that effects reported here are relative to a within-subjects control condition, permitting a discussion of how the post-hypnotic suggestion affects ex-Gaussian parameters.

## Discussion

Ex-Gaussian analysis of the data from Parris et al. revealed that the post-hypnotic word blindness suggestion takes its effect in μ, the modal portion of the RT distribution, in the present data set. It has been argued that μ is where response competition is mainly registered (Kane and Engle, 2003; Steinhauser and Hübner, 2009). An effect of the suggestion on μ is therefore indicative of a mechanism that operates over response competition instead of inducing word blindness (see also Augustinova and Ferrand, 2012). Such an account can also explain why facilitation would not be affected by the suggestion since facilitation does not involve response competition. On this interpretation, word reading itself has not been suppressed by the suggestion, but rather response conflict dealt with more effectively. Whilst one has to be careful when attributing cognitive processes to ex-Gaussian parameters, converging evidence supports the notion that the suggestion operates over the response competition mechanism. A similar pattern to that observed in μ was observed in σ, the measure of the standard deviation in the modal portion of the distribution. Finally, the present data were inconclusive with regards to the effect of the suggestion on facilitation in all ex-Gaussian parameters and with regards to the effect of the suggestion on interference in τ. However, despite being inconclusive about the effect of the suggestion in τ, the present results indicate that any remaining interference in the algebraic mean data is likely to be in the tail of the RT distribution; a finding that highlights the importance of the criteria set for outlier selection.

A further important finding from the present analysis supports the utility of ex-Gaussian analysis in revealing effects that might otherwise be thought of as absent (Heathcote et al., 1991; Balota and Yap, 2011) and shows how distributional analysis increases the sensitivity of detecting effects on underlying processes. Whilst Parris et al. reported a modulating influence of RSI on algebraic means, with no effect for long RSI, the analysis of ex-Gaussian parameters showed that the suggestion did indeed influence interference when RSI was long (3500 ms; roughly equivalent to that employed by Raz and colleagues) in our data. As in the short RSI condition the suggestion resulted in a distributional leftward shift in the long RSI condition. The effect on interference in the long RSI condition is numerically smaller than in the short RSI condition, which accounts for the need for the more sensitive analysis to detect it. The finding observed here indicates that there were indeed trials on which the suggestion took an effect in the long RSI condition, but that the effect is harder to detect at longer RSIs.

Problematic for an account of the word blindness suggestion effect based on response competition is that it would predict that facilitation could never be affected by the suggestion since it is likely that congruent trials do not involve response competition. Contrary to this prediction, Raz et al. (2002) observed an elimination of Stroop facilitation in their study, but this effect has rarely been replicated (see Table 1). More research is needed to elucidate how, when and how effectively the word blindness suggestion operates, especially given the potential differences in how Stroop interference and facilitation are produced (MacLeod and MacDonald, 2000; Kane and Engle, 2003; Goldfarb and Henik, 2007).

The aim of the present paper was to apply the ex-Gaussian function to the data reported by Parris et al. (2012). Ex-Gaussian analysis revealed that: (1) The suggestion takes its effect in μ in the present data, which converging evidence indicates is the result of the suggestion operating over response competition mechanisms; and, (2) Contrary to the data reported by Parris et al. there was an effect of the suggestion in the long RSI condition. Overall, the reanalysis of the data from Parris et al. (2012) supports the utility of ex-Gaussian analysis in revealing effects that might otherwise be thought of as absent (Heathcote et al., 1991; Balota and Yap, 2011) and shows how distributional analysis increases the sensitivity of detecting effects on underlying processes.

### Conflict of interest statement

The authors declare that the research was conducted in the absence of any commercial or financial relationships that could be construed as a potential conflict of interest.
